# Parasitoid wasp venom re-programs host behavior through downmodulation of brain central complex activity and motor output

**DOI:** 10.1242/jeb.245252

**Published:** 2023-02-13

**Authors:** Amit Rana, Michael E. Adams, Frederic Libersat

**Affiliations:** ^1^ Department of Life Sciences and Zlotowski Center for Neurosciences, Ben Gurion University of the Negev, Beer Sheva, Israel; ^2^ Departments of Entomology and Molecular, Cell, and Systems Biology, University of California, Riverside, CA 92521, USA

**Keywords:** Venom, Central complex, Cockroach, Descending control, Locomotion, Parasitoid wasp

## Abstract

The parasitoid wasp *Ampulex compressa* hunts down its host, the American cockroach (*Periplaneta americana*), and envenomates its brain to make it a behaviorally compliant food supply for its offspring. The primary target of the wasp sting is a locomotory command center called the central complex (CX). In the present study, we employ, for the first time, chronic recordings of patterned cockroach CX activity in real time as the brain is infused with wasp venom. CX envenomation is followed by sequential changes in the pattern of neuronal firing that can be divided into three distinct temporal phases during the 2 h interval after venom injection: (1) reduction in neuronal activity for roughly 10 min immediately after venom injection; (2) rebound of activity lasting up to 25 min; (3) reduction of ongoing activity for up to 2 h. Long-term reduction of CX activity after venom injection is accompanied by decreased activity of both descending interneurons projecting to thoracic locomotory circuitry (DINs) and motor output. Thus, in this study, we provide a plausible chain of events starting in the CX that leads to decreased host locomotion following brain envenomation. We propose that these events account for the onset and maintenance of the prolonged hypokinetic state observed in stung cockroaches.

## Teaser

**Summary:** Wasp venom has a direct effect on brain activity and associated changes in descending activity and motor output that are likely to account for the hypokinetic state observed in envenomated cockroaches.

## INTRODUCTION

The jewel wasp *Ampulex compressa* hijacks the brain of its host animal, the American cockroach *Periplaneta americana*, rendering it a compliant food source for its offspring (Haspel et al., 2005). To achieve this goal, the wasp stings the cockroach in the first thoracic ganglion to disable its front legs (Gal and Libersat, 2010; Haspel and Libersat, 2003; Libersat, 2003; Moore et al., 2006) and subsequently in the head ganglia to induce a complex behavioral sequence (Libersat, 2003). Shortly after the head sting is employed, the cockroach initiates a phase of compulsive grooming lasting for ∼30 min (Libersat, 2003; Weisel-Eichler et al., 1999), followed by a long-lasting hypokinetic state in which the cockroach shows little motivation to initiate or maintain walking behavior (Libersat, 2003). However, the effect of the wasp venom is unique in the sense that it selectively affects some motor behaviors, while sparing others. For example, walking and escape running are severely affected, while other behaviors, such as the righting response, flying and swimming, remain unaffected (Gal and Libersat, 2008).

Using radio-labeled wasps, Haspel et al. (2003) found that the wasp injects venom in both cephalic ganglia, the cerebral ganglia (CRG; supra-esophageal ganglia) and the gnathal ganglia (GNG; previously named the subesophageal ganglia). When measuring levels of radioactivity in head ganglia, these authors found that the bulk of the venom (∼80%) is injected in the CRG and the remaining 20% in the GNG. Moreover, using light microscope autoradiography, they also showed that the epicenter of the venom volume was in and around a brain region known as the central complex (CX) (Haspel et al., 2003; Turner-Evans and Jayaraman, 2016). This multi-layered structure consists of three distinct sub-regions: the proto-cerebral bridge (PB), the fan-shaped body (FB) and the ellipsoid body (EB). The CX functions as a sensory and pre-motor command center in the insect brain. As a sensory center, it is responsible for processing information related to polarized light (Heinze and Homberg, 2007; Heinze et al., 2009), antennal deflection (Ritzmann et al., 2008) and nociception (Rana et al., 2022). As a premotor command center, the CX is directly involved in locomotion. For instance, the CX neuronal firing rate is correlated with the stepping rate and electrical stimulation of CX leads to an increase in the stepping rate (Bender et al., 2010). Moreover, several CX neurons exhibit an increased firing rate preceding onset onset of turning behavior (Guo and Ritzmann, 2013).

Hence, the CX functions as a sensory-motor hub to control various behaviors. Descending interneurons (DINs) originating from cephalic ganglia project to and beyond thoracic ganglia (Okada et al., 2003; Staudacher and Staudacher, 1998) and columnar neurons in the CX project to the lateral accessory lobe (LAL) (Heinze and Homberg, 2008), which appears to select DIN activity projecting to thoracic motor centers (Wolff and Strausfeld, 2015). Previous studies have reported that ∼65% of DINs send axons through neck connectives ipsilateral to their soma (Namiki et al., 2018), thus connecting the cephalic ganglia to the thoracic ganglia. Each thoracic ganglion controls a pair of legs, respectively. Recordings from the coxal muscle of the metathoracic leg revealed that slow motoneurons (Ds) are recruited for posture and slow walking (Emanuel and Libersat, 2017; Emanuel et al., 2020; Watson and Ritzmann, 1998a), while fast motoneurons (Df) are recruited for escape responses (Watson and Ritzmann, 1998b). Although the primary target for the wasp sting to the brain is the CX, the ultimate consequence is reflected as suppression of Ds motor activity and the virtual absence of Df activity (Emanuel and Libersat, 2017). In addition, Emanuel and Libersat (2017) found that Ds motoneuron activity in the coxal muscle of metathoracic leg is drastically reduced following procaine injection into the CX (Emanuel and Libersat, 2017).

Previous studies have revealed involvement of both GNG and CRG in venom-induced locomotory hypokinesia. Earlier studies showed that the wasp actively targets the GNG during the head sting and that time spent searching for GNG is prolonged following its extirpation (Gal and Libersat, 2010). Similarly, the head sting is dramatically prolonged, often by a factor of 10 or more following CRG removal, showing that the wasp actively searches for the CRG as well during the sting sequence (Gal et al., 2014). Hence, during the head sting the wasp actively targets not only the GNG, but also the CRG, and this is substantiated by evidence for deposition of radiolabeled venom in both ganglia following the head sting (Haspel and Libersat, 2003). These previous studies thus demonstrate unequivocally that the wasp targets both cephalic ganglia during head envenomation to produce locomotory hypokinesia.

Abundant evidence likewise demonstrates that both cephalic ganglia are actively involved in control of locomotion. Activity of the GNG leads to initiation but primarily to coordination of central pattern generators in the thoracic ganglia contributing to leg motor output (Gal and Libersat, 2006; Knebel et al., 2018a,b). Our previous studies showed that injection of either procaine or wasp venom in the GNG decreases the propensity for spontaneous and evoked walking in cockroaches (Gal and Libersat, 2010). However, procaine as a voltage-dependent sodium blocker not only suppresses activity in the GNG, but as a side effect, also shuts down descending traffic from the CRG. In fact, procaine injection into the CX in the CRG abolishes or drastically shortens spontaneous walking bouts (Kaiser and Libersat, 2015). Thus, while the envenomation of the GNG is likely sufficient for suppression of locomotory activity, injection by the wasp of a ∼4-fold larger quantity of venom into the CRG, a known command center for walking, may be critical for establishment of the hypokinetic state.

With this in mind, we hypothesized that *Ampulex* venom modulates excitability of the CX with decisive consequences for the descending control of walking. In this study, we demonstrate that decreased CX excitability is reflected in reduced DIN input to thoracic ganglia. This could account for the inability of stung cockroaches to initiate spontaneous walking or escape running. Our findings identify a chain of events starting with changes in CX neuronal activity for up to 2 h after venom injection (time required for the onset of hypokinesia), leading to suppression of DIN activity, and ultimately depression of thoracic motoneuron activity. We propose that linkage of these events plays an important role in wasp venom-induced hypokinesia.

## MATERIALS AND METHODS

### Animals

All experiments were performed on adult male cockroaches [*Periplaneta americana* (Linnaeus 1758)] that were raised in crowded conditions in plastic containers (50×50×70 cm) under a 12 h:12 h light:dark cycle at 26°C. Water and food (cat chow) were provided *ad libitum*. Adult female wasps [*Ampulex compressa* (Fabricius 1781)] were raised at 25–30° C in relative humidity of 50% or above under a 12 h:12 h light:dark photoperiod. Wasps were provided water and honey *ad libitum*. The experiments performed comply with the Principles of Animal Care, National Institutes of Health (NIH) publication no. 86-23 revised in 1985, and with the current laws of the State of Israel.

### Venom collection

Wasps were anesthetized by chilling on ice for 3 min. Wings were ablated to limit the mobility of the wasp upon recovery. Venom obtained by a milking procedure described previously (Kaiser and Libersat, 2015) was collected using a nanovolumetric injector (Drummond Nanoinject II). The needle of the nanoinjector was kept close to ice throughout the process. The milking procedure did not take more than 25 min.

### Electrophysiology recordings

To estimate the holistic effect of wasp venom on the cockroach central nervous system (CNS), we performed various extracellular electrophysiological experiments at multiple CNS regions involved in motor control. Two-channel mono-polar electrodes were used to record chronic CX activity, hook electrodes were used to record DINs activity and bi-polar electrodes were employed to record EMG activity from the coxal muscles of metathoracic leg. Details of all the electrophysiological procedures are described below.

#### Chronic CX neuronal activity

To estimate the effect of CX envenomation, chronic CX neuronal activity was recorded (*n*=35) using a TDT Rz5 bio-amp processor amplifier (Tucker Davis Technologies, United States) suitable for multichannel recordings. A custom-built two-channel mono-polar electrode (Formvar coated 37 µm NiCr from A-M systems) was built and inserted in the central complex using a micromanipulator, as described previously (Guo et al., 2014; Rana et al., 2022). A ground electrode (75 µm silver wire from A-M systems) was inserted into the hemolymph through the pronotum (Rana et al., 2022). Before the experiment, the cockroach was anaesthetized by chilling on ice for 5 min. Once anaesthetized, wings were clipped using fine scissors and the animal was secured on a horizontal surface by insect pins. To reduce hemolymph pulsations entering the head capsule, a horseshoe-shaped pin was placed around the neck. Subsequently, fine scissors were used to remove most of the cuticle from the head capsule, including the antennal lobe and ocelli. Furthermore, the head capsule was cleared of excessive fat and trachea using fine forceps for better visualization of the CRG. A wax-coated metal wire platform was placed beneath the CRG to enhance stability of the recording preparation and to limit its movement during the injection process (Rana et al., 2022; Saha et al., 2013).

CX recordings were initiated upon the detection of spontaneous spikes (Rana et al., 2022). Since CX neurons generally are multimodal and respond to light stimuli, we performed a preliminary assessment of electrode location by testing for sensitivity to light-ON and light-OFF stimuli as a first assessment of the location of our electrode inside the CX (Ritzmann et al., 2008). Subsequently, a nanovolumetric injector (Drummond Nanoinject II) was used to deliver a total of 27 nl of crude venom into the CX in 3 aliquots of 9 nl (Rana et al., 2022). One hour after venom injection, neuronal responses to light stimuli were tested again. To ensure viability of the preparation over a period of 2 h, we used two control groups. In the first control group, procaine was injected in the CX, while a second control group was injected with saline instead of venom using the same protocol.

#### DIN traffic from head ganglia to prothoracic ganglion

To estimate the effect of the wasp sting on DIN traffic in tethered unstung (*n*=7) and stung (*n*=8) cockroaches, we performed extracellular hook electrode recordings from the neck connectives. In this set of experiments, unstung cockroaches served as a control group. Access to the dorsal side of the head capsule for venom injection while recording from the neck connectives presents extreme difficulties. Hence, in this set of experiments, we used a natural wasp sting for CX envenomation instead of a milked venom injection. Recordings from stung cockroaches were initiated 4–6 h after the wasp sting. To establish a stable recording, the anaesthetized animal was pinned ventral side up on the horizontal platform after ablating the legs and wings. A mid-ventral incision was made in the transparent cuticle between the gnathal ganglion (GNG) and prothoracic ganglion. The nerve was carefully lifted and placed on hook electrodes by a fire-polished glass probe. The nerve was crushed posterior to the recording site to remove ascending activity from thoracic interneurons. Neuronal responses to antennal touch were checked and animals that failed to generate a response were discarded. Similarly to the CX recordings, spike sorting was also performed on DIN activity and only units that could be extracted on the basis of waveform analysis were used for further analysis.

#### EMG activity from the coxal muscle of the hind leg

To establish the relationship between CX neuronal activity and onset of the hypokinetic state, we recorded activity from the coxal muscle of the metathoracic leg (*n*=6 units from 6 animals) along with chronic CX neuronal activity (*n*=9 units from 6 animals). To do so, the cockroach was anaesthetized by chilling on ice for 5 min, then fastened ventral side up on a horizontal platform. A custom-built bipolar electrode was inserted into the coxal muscle. Upon detection of slow motoneuron (Ds) activity, the position of the implanted electrode was secured by wax. The animal was anaesthetized again by placing on ice for CX dissection as described in the chronic CX recording section.

### Light stimulus

To test the CX neuronal response to the light stimulus, a custom-built platform with 5 blue LEDs was placed 10 cm away from the animal. All the lights in the recording setup were turned off to ensure complete darkness. Then a 500 ms long stimulus was delivered using an isolated pulse stimulator (A-M systems, model 2100). In each of the conditions, i.e. before and after the venom injection, 30 trials of light stimuli were presented with an inter-trial interval (ITI) of 20 s.

### Histology

**Fig. 1.**
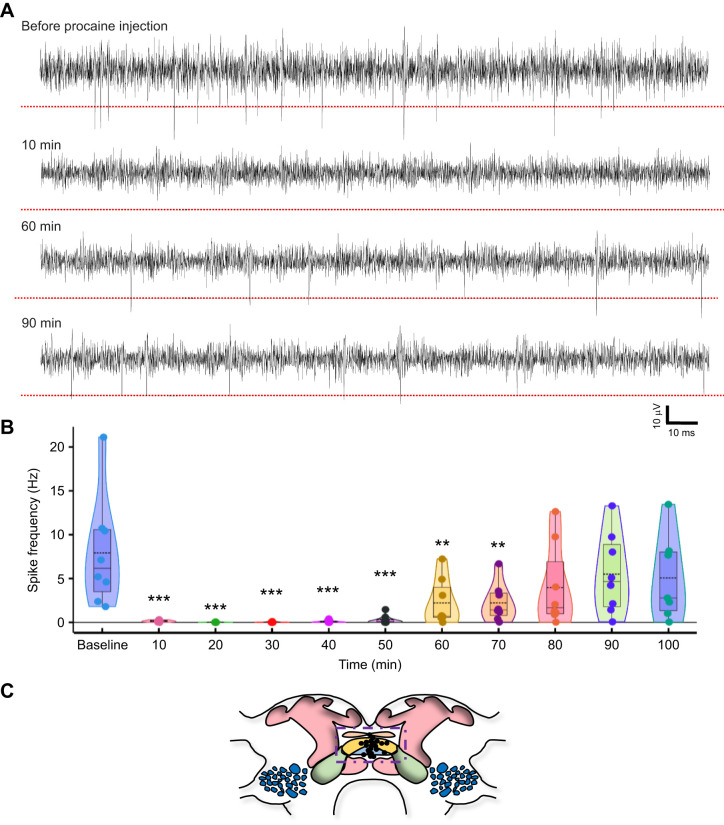
**Injection of procaine causes a transient decrease in central complex (CX) activity.** (A) Representative example trace of spontaneous neuronal activity in the CX at various time intervals before and after procaine injection. Procaine injection inhibits spontaneous activity of the CX immediately after injection. Spontaneous activity begins to recover 60 min after injection. (B) Spike frequency changes following procaine injection binned in 10 min intervals. Violin plot showing quantitative analysis of spontaneous CX neuronal activity. Baseline activity is calculated as an average spike frequency 20 min prior to injection, and spike frequency was binned in 10 min time intervals up to 100 min after the injection to estimate post-injection neuronal activity. Solid circles represent an individual unit (*n*=8 from 5 animals; mean±s.e.m. firing rate, *P*-value and *t*-value in Table S1, one-way RM ANOVA, multiple comparisons versus baseline using the Bonferroni *t*-test). The boxes represent the 25th and 75th percentiles of the distribution of values. Within each box, dotted black lines represent mean spike frequency, horizontal black lines denote median values. Vertical extending lines (whiskers) denote adjacent values indicating 95% range significance as indicated with asterisks (**P≤0.01; ***P≤0.001). (C) Diagram of the CX region (dashed purple box) drawn after Varga et al. (2017) (protocerebral bridge, pink; fan-shaped body, orange structure; ellipsoidal body, blue). Mushroom bodies are marked in pink, lateral accessary lobe in green and antennal lobe in blue. Black dots indicate tip position of electrode estimated from histological analysis.

To mark the location of the electrode during chronic CX recordings, we coated the electrode with DiI (1,1-dioctadecyl-3,3,3,3-tetramethylindocarbocyanine iodide; sc391087, Santa Cruz Biotechnology, Inc.). In addition to DiI, an electric current of 10 µA was passed through the electrodes for 5 s to induce a lesion at the end of the experiment (Rana et al., 2022). Once the recording was completed, the cockroach was decapitated and the head was fixed overnight in 10% formalin. The head was cleaned using saline to remove excess formalin. Subsequently, the CRG was dissected from the head capsule and embedded in 6% agarose. An Olympus BH-2 fluorescence microscope was used to detect the trace of DiI or current-induced lesion in the 60 µm thick sections of agarose embedded CRG, and the electrode tip position was identified and marked (Fig. 1C).

### Analysis and statistics

Electrophysiological data obtained from the extracellular recordings was analyzed using a custom-built Spike2 script (V5.05, Cambridge Electronic Design). Potential spikes were detected from each recording by a manually defined threshold (twice in the amplitude of background noise). These potential spikes were sorted on the bases of their unique waveform (spike sorting). Spikes were considered to belong to the same unit if 70% or higher similarity was found in their waveform. Furthermore, principal component analysis was employed for feature detection and clustering. We also verified with inter-spike-interval (ISI) analysis that each cluster of spikes come from a single neuron (ISI of only 0.2% of spikes was <2 ms). Each cluster of spikes was considered as a single unit. Once single units were identified, the timing of each spike was also estimated. To estimate the effect of injection (procaine or venom) on spontaneous CX activity, the neuronal firing rate (spike frequency) was determined. Using this spike frequency, baseline activity and neuronal activity after injection were calculated. Spike frequency was averaged for 20 min prior to the injection to estimate baseline activity, and spike frequency was binned in 10 min time windows up to 2 h after injection to estimate post-injection neuronal activity. It is important to mention that with the custom made two channel monopolar electrodes, we were able to record only a subset population of CX neurons in each recording. However, to keep the results straightforward for the reader, we have described this subset population of the CX simply as ‘CX activity’. Furthermore, to test the evoked activity of single units, the neuronal response to a 500 ms light stimulus was tested. The timing of each isolated unit across 30 trials of light stimulus before and after venom injection were used to plot the peri-stimulus time histogram (PSTH) evoked by light stimuli. The PSTH plot begins 500 ms before onset of the light stimulus (baseline activity) and lasts until 1500 ms after stimulus onset (light stimulus-evoked activity). The 2 s recordings of evoked neuronal activity of individual CX units were sampled in 100 ms as well as 250 ms time bins for each stimulus type. To calculate the response duration to light stimuli, the period of significantly increased evoked activity as compared with the baseline activity was calculated from the PSTH data. Baseline activity was calculated as an average of neuronal activity 500 ms prior to light stimuli. To calculate total evoked activity, neuronal spikes were summed within 1 s from stimulus onset. To determine latency of response, we measured the time difference between stimulus onset and peak neuronal activity, which in turn was calculated as the maximum number of spikes within a 20 ms time bin after stimulus onset.

Statistical significance was evaluated using SigmaPlot 13.0 software and MATLAB (R2018a). For graphical representation, MATLAB and online plotting studio was used (https://chart-studio.plotly.com/). Normal distribution of data was tested using the Shapiro–Wilk test; if data were found not to be normally distributed, non-parametric statistical tests were used. Repeated measures (RM) ANOVA on ranks was employed to estimate the significance in the duration of response to light stimuli. Dunnett's test was performed to compare evoked firing rate of light responsive units to baseline firing rate. Statistical significance for light responsive units before and after venom injection was determined using paired *t*-tests (two-tailed). Pairwise comparison of spontaneous activity for injection recording (procaine and venom) was performed using one-way RM ANOVA, followed by multiple comparisons versus control group (baseline) using the Bonferroni *t*-test. Statistical significance in the leg EMG activity was established using the Wilcoxon signed-rank test. Pearson correlation analysis was employed to test the correlation between CX activity (*n*=9 units from 6 animals) and EMG activity (*n*=6 units from 6 animals). To measure the strength of DINs activity, mean firing rate for both stung and unstung cockroaches was calculated. Mean firing rate was determined as the average number of spikes within a 10 min recording window. The distribution normality of DINs data was tested using the Shapiro–Wilk normality test, and non-normally distributed data was tested using the Mann–Whitney rank sum test.

## RESULTS

### Procaine injection into the CX leads to reversible inhibition of spontaneous activity

In this study, our experimental strategy involves micropipette impalement of the CX for direct injection of venom and recording of neuronal activity over a period of 2 h. We first controlled for potential non-specific effects of our experimental protocol, since injection into the CX and total duration of the experiment may affect stability of the recording and viability of the preparation. Thus, to test the viability of animal preparations for venom injection protocol, we injected procaine (a reversible Na^+^ channel inhibitor) into the CX and recorded its activity over a period of 2 h (Fig. 1A). The CX activity was used to estimate spike frequency (neuronal firing rate per second). Baseline activity was calculated as an average spike frequency 20 min prior to injection. Spike frequency was binned in 10 min time intervals up to 100 min after the injection to estimate post-injection neuronal activity. As expected, procaine induced a drastic decrease in CX neuronal activity briefly after the injection (Fig. 1A,B; mean±s.e.m. firing rate: 0.15±0.04 Hz, *P*<0.001, *t*=5.019, one-way RM ANOVA, multiple comparison against control using Bonferroni *t*-test) compared with baseline activity (7.92±2.22 Hz). This reduction in spontaneous CX neuronal activity was maintained for ∼50 min after injection (Fig. 1B, Table S1). From then on, neuronal activity began to recover and by 80 min, it returned to a level that was statistically indistinguishable from the pre-injection baseline level (Table S1). Moreover, neuronal activity continued to recover over time and by 90 min post-injection reached a level that was identical to the pre-injection baseline (Fig. 1B; 5.06±1.83 Hz, *P*=1.000, *t*=1.565). These data demonstrate complete recovery of spontaneous activity of CX neurons following our injection protocol and confirm viability of the recording preparation during the period of investigation. It is important to mention that while procaine injection into the CX induces a reversible inhibition of the neuronal activity, saline injection into the CX had neither short-term nor long-term modulatory actions on CX neuronal activity (Fig. S1).

### Wasp venom has a distinctive time-dependent effect on spontaneous activity of CX

**Fig. 2.**
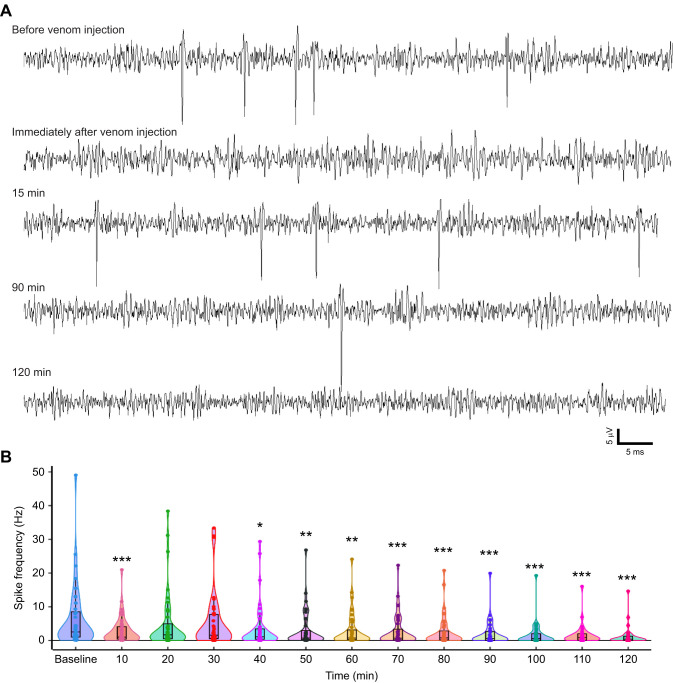
**Venom injection induces a distinctive time-dependent effect on the spontaneous activity of the CX.** (A) Representative example of CX neuronal activity at various time intervals before and after venom injection. CX spontaneous activity disappears immediately after venom injection. This silencing of CX activity is transient and neuronal activity is restored after 10 min and remains elevated for the next 20 min, only to decrease again over time. (B) Violin plot showing quantitative analysis of spontaneous CX neuronal activity. Baseline activity is calculated as an average spike frequency 20 min prior to injection, spike frequency was binned in 10 min time windows up to 120 min after the injection to estimate post-injection neuronal activity. Solid circles represent spike frequency in each of the animals at the corresponding time bin (*n*=35 units from 21 animals; mean±s.e.m. firing rate, *P*-value and *t*-value in Table S2, one-way RM ANOVA, multiple comparisons versus baseline using the Bonferroni *t*-test). The boxes represent the 25th and 75th percentiles of the distribution of values. Within each box, dotted black lines represent mean spike frequency, horizontal black lines denote median values. Vertical extending lines (whiskers) denote adjacent values indicating 95% range; *P≤0.05; **P≤0.01; ***P≤0.001, significantly different from the pre-venom injection baseline.

To estimate the impact of wasp venom on spontaneous CX activity, spike frequency was determined to calculate baseline and post-injection neuronal activity in a similar manner as described for procaine injection (Fig. 2A). We used one-way RM ANOVA, multiple comparisons against control (baseline) using Bonferroni *t*-test (Fig. 2B) to quantify the spontaneous CX activity. We found that there was a significant decrease in spontaneous activity immediately following venom injection (mean±s.e.m. firing rate for 10 min: 2.89±0.48 Hz, *P*=0.001, Fig. 2B) compared with baseline activity (6.32±1.65 Hz). Following this decrease, CX neuronal activity rebounded for the next 20 min (Table S2, Fig. 2B). However, this elevated firing rate was short-lived and thereafter, CX activity continued decreasing over time. Reduction in spontaneous activity became significant 30 min post-venom injection (Table S2, *P*=0.043, *t*=2.517) and remained suppressed for the entire recording window up to 2 h (Table S2).

### Wasp venom diminishes the CX response to light

**Fig. 3.**
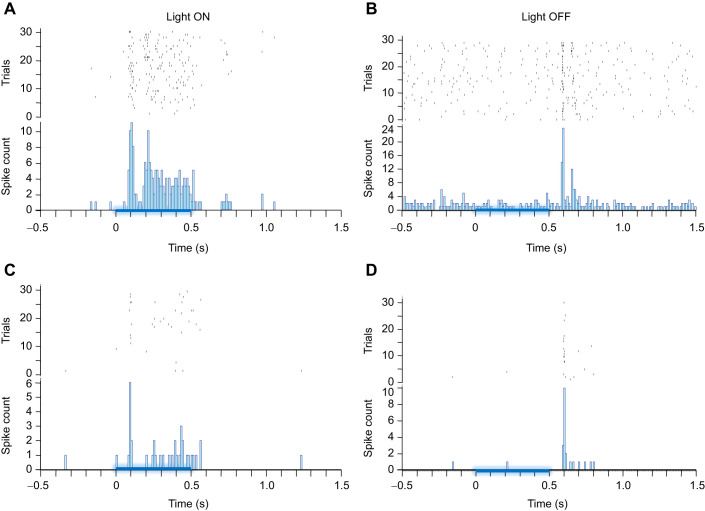
**Both light-ON and light-OFF units respond with elevated activity within their response window, but the response is diminished after venom injection.** (A,C) Representative example of raster plots and post-stimulus time histograms (PSTHs) of an individually identified light ON unit before (A) and after (C) venom injection. (B,D) Raster plots and PSTHs for light OFF units before (B) and after (D) venom injection. In a single experiment, data were collected from 30 trials before and after venom injection. The blue line on the *x*-axis represents the time course of the light stimulus.

**Fig. 4.**
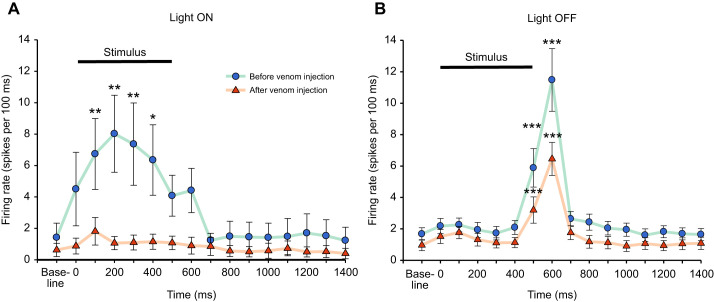
**Wasp venom diminishes the CX response duration to light stimuli.** (A) Peri-stimulus time histograms for light ON unit response (*n*=8 units from 5 animals) before and after venom injection. The solid black line represents the duration of the stimulus. Light ON unit activity increases and remains elevated for the entire 500 ms stimulus duration as compared with the baseline activity. The mean CX response decreases after venom injection to light ON stimulus and no time bin shows any increase in evoked activity after venom injection. (B) Peri-stimulus time histograms for light OFF unit response (*n*=8 units from 5 animals) before and after venom injection (*n*=24 units from 15 animals). Light OFF unit response duration is ∼200 ms from the stimulus offset. No change in response duration occurs after venom injection but the mean CX response is reduced by half of its pre-venom injection level. Data points represent means±s.e.m. for all units identified in different animals, each animal is presented as 30 trials of light stimulus before and after the venom injection. *P≤0.05; **P≤0.01; ***P≤0.001, significant difference in the light-evoked responses before and after venom injection using Friedman RM analysis on ranks followed by multiple comparisons versus control (Dunnett's method).

To assess actions of wasp venom on sensory input to CX neurons, we recorded CX responses to brief 500 ms light pulses. We identified two distinct types of units based on responses to light stimuli: (1) light ON units that respond within the light stimulus window (Figs 3A and 4A, *n*=8 units); (2) light OFF units that respond briefly after the stimulus offset (Figs 3B and 4B, *n*=24 units). We further analyzed these light-responsive units to estimate parameters such as stimulus–response duration, peak neuronal response, total evoked activity and latency of the evoked response.

Our results indicate that response duration varies depending on the type of unit (light ON or light OFF). Before the venom injection, light ON unit activity increased and remained elevated for the entire 500 ms stimulus duration compared with the baseline (Fig. 4A, green line; Friedman RM analysis on ranks, multiple comparisons using Dunnett's method, single unit raster and PSTH are shown in Fig. 3A). In contrast, the light OFF unit response duration was ∼200 ms from the stimulus offset (Fig. 4B, green line; Fig. 3B). Furthermore, venom injection into the CX reduced the activity of each type of light-responsive unit. For light ON units, venom injection completely suppressed the neuronal evoked response (Fig. 4A, orange line); no time bin showed any significant difference in evoked activity compared with baseline activity (Fig. 4A). By contrast, for light OFF units, the duration of response remained the same before and after the venom injection (200 ms). However, strength of evoked neuronal activity decreased greatly after venom injection (Fig. 4B, orange line).

**Fig. 5.**
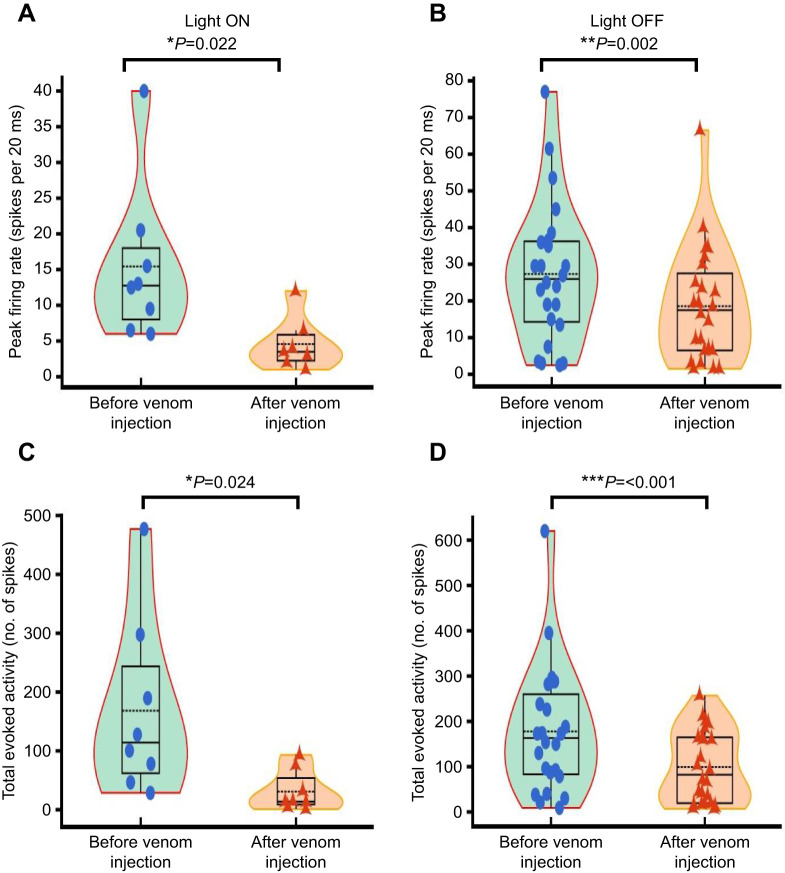
**Wasp venom desensitizes the CX response to the light stimulus.** (A) Peak firing rate of light ON units decreases after venom injection (*n*=8 units from 5 animals; *t*=3.066 with 6 degrees of freedom, paired *t*-test). (B) Peak firing rate of light OFF units decreases after venom injection (*n*=24 units from 15 animals; *t*=3.398 with 23 degrees of freedom, paired *t*-test). (C) Total evoked activity decreases after venom injection for light ON units (*n*=8 units from 5 animals, *t*=2.868 with 7 degrees of freedom, paired *t*-test). (D) Total evoked activity decreases after venom injection for light OFF units. Venom injection suppresses the CX response to light OFF stimulus (*W*=−260.000, *T*^+^=20.000, *T*^−^=−280.000, *Z*-statistic based on positive ranks=−3.715, Wilcoxon signed rank test, *n*=24 units from 15 animals). Total evoked activity is determined as the neuronal spikes within 1 s from stimulus onset and peak firing rate is calculated as the maximum number of spikes within a 20 ms time bin after stimulus onset within the response duration window. The violin plot combines box plot and data points to describe modulation of light-evoked CX activity due to wasp venom. The boxes represent the 25th and 75th percentiles of each group's distribution of values. Within each box, dotted black lines represent mean value, horizontal black lines denote median values; whiskers denote adjacent values indicating 95% range (*P≤0.05; **P≤0.01; ***P≤0.001).

Furthermore, to estimate the strength of evoked response to the light stimulus, we calculated total evoked activity by summing neuronal spikes within 1 s of stimulus onset. Total evoked activity decreased significantly after venom injection regardless of stimulus type (Fig. 5C,D). For light OFF units, total evoked activity decreased by ∼45% after the venom injection compared with before injection (Fig. 5D). Likewise, in the case of light ON units, effects of venom injection were even more impressive, suppressing total evoked activity by 80% (Fig. 5C, paired *t*-test).

### Latency and peak neuronal response

To estimate speed of information processing and to estimate recruitment of additional neuronal processes by sensory light stimuli, we calculated latency of response by estimating the time difference between stimulus onset and peak neuronal activity, which in turn was calculated as the maximum number of spikes within a 20 ms time bin after stimulus onset within the PSTH time window. We identified a clear reduction in peak neuronal responses for both types of light-responsive units. For light ON units (*n*=8 units from 5 animals), peak neuronal responses decreased by more than 50% after venom injection compared with before injection (Fig. 5A). Likewise, for light OFF units (*n*=24 units from 15 animals), peak neuronal responses decreased by ∼30% after venom injection (Fig. 5B). In addition, wasp venom also diminished speed of sensory processing as estimated from response latency. For light ON units, response latencies trended toward increase after venom injection (mean±s.e.m. latency: 195.95±40.05 ms, *n*=8 units from 5 animals) compared with before injection (125.25±23.23 ms; graph not shown, *n*=8 units from 5 animals). However, this increase in latency ws not statistically significant (*P*=0.334; two-tailed *t*=−1.049 with 6 degrees of freedom, paired *t*-test). Likewise for light OFF units, response latencies increased significantly after venom injection (601.35±4.26 ms, *P*=0.042, *W*=46,000, *T*^+^=56,000, *T*^−^=−10.000, *Z*-statistic based on positive ranks=2178, Wilcoxon signed rank test; *n*=24 units from 15 animals) compared with before injection (592.61±3.92 ms; graph not shown; *n*=24 units from 15 animals).

### Wasp venom reduces DIN traffic from the cerebral ganglia

**Fig. 6.**
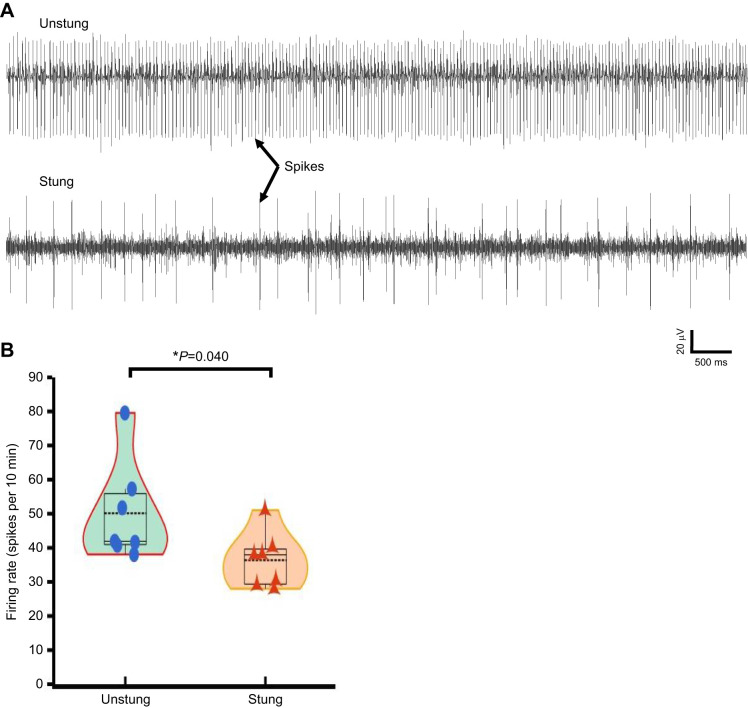
**CX envenomation reduces DIN traffic.** (A) Representative examples of descending interneuron traffic in unstung and stung cockroaches. (B) Descending interneuron firing rate is significantly reduced in stung animals (*n*=8) compared with the controls (*n*=7; Mann–Whitney *U*-statistic=10.000, Mann–Whitney rank sum test). Descending interneuron firing rate is determined as the average number of spikes within 10 min recording window. The violin plot combines box plot and data points to describe the reduction in the DINs traffic after the venom injection. The boxes represent the 25th and 75th percentiles of each group's distribution of values. Within each box, dotted black line represents the mean value, horizontal black lines denote median values; whiskers denote adjacent values indicating 95% range (*P≤0.05).

The aforementioned results provide substantial evidence that wasp venom reduces both spontaneous neuronal activity as well as evoked neuronal activity of the CX over time. Considering the importance of the CX as a hub for information processing and transfer to thoracic locomotory centers, we then focused on investigating the effect of CX envenomation on DIN activity recorded from neck connectives between the GNG and prothoracic ganglia from both unstung and stung cockroaches (Fig. 6A). In this experiment, unstung cockroaches serve as a control group. Similarly to the CX recordings, spike sorting was also performed on DIN activity and only units that could be extracted on the basis of waveform analysis were used for the further analysis. Spontaneous DIN activity of individually identified units was tested for mean firing rate in stung (*n*=8) and unstung (*n*=7) animals. Recordings from stung animals were established 4–6 h after the wasp sting. Each stung animal was tested for hypokinesia before the recording. Envenomation of CX led to a 33% reduction in DIN traffic. Mean firing rate estimated in stung cockroaches was significantly lower than mean firing rate in unstung animals (Fig. 6B).

### Modulation of CX activity is followed by reduction of leg muscle activity

**Fig. 7.**
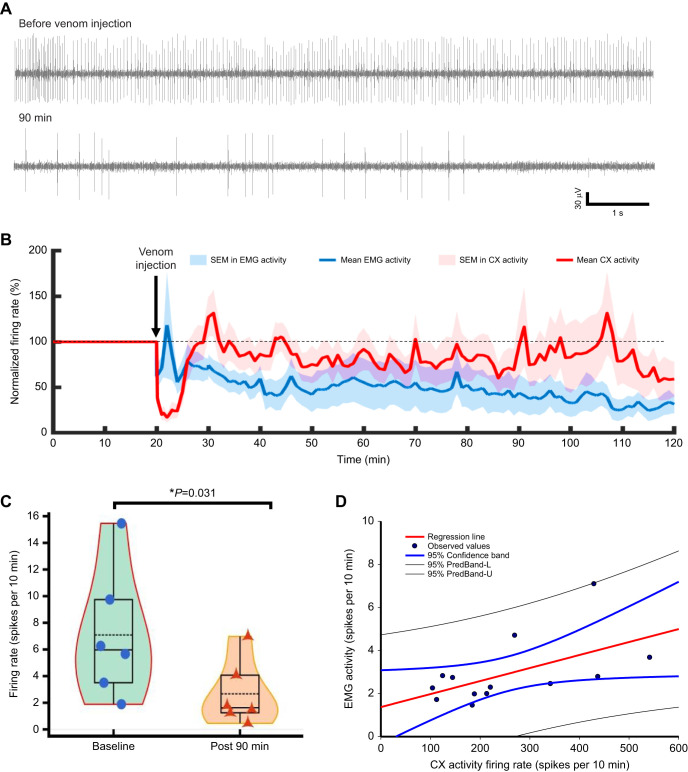
**CX envenomation reduces the leg EMG activity in a top-down manner.** (A) Representative example of EMG activity before venom injection and 90 min after venom injection. (B) Normalized CX activity (red line with red shaded region) as well as normalized leg EMG activity (blue line with blue shaded region) reduces after venom injection as a function of time. Solid lines represent mean firing rate and the shaded region indicates s.e.m. Black dotted line represents the 100% normalized activity level. (C) Ds activity is reduced significantly 90 min post venom injection as compared with baseline activity (*n*=6, *W*=−21,000, *T*^+^=0.000, *T*^−^=−21,000, *Z*-statistic based on positive ranks=−2.201, Wilcoxon signed-rank test). The violin plot combines the box plot and data points to describe the reduction of leg EMG activity after the venom injection. The boxes represent the 25th and 75th percentiles of each group's distribution of values. Within each box, dotted black lines represent the mean value, horizontal black lines denote median values. Vertical extending lines (whiskers) denote adjacent values indicating 95% range, significance is indicated with asterisks. (D) Reduction of CX activity positively correlates with reduction of leg EMG activity after venom injection. Solid circles represent observed data points, red line represents the regression line calculated from the linear polynomial equation and blue lines represent the 95% confidence band, black lines represent the 95% prediction band; Pearson correlation analysis, *r*=0.560, *P*=0.046.

To estimate whether reduction in CX activity is correlated with reduced thoracic motor activity, we recorded activity of the slow coxal depressor motoneuron (Ds) innervating the metathoracic leg (Fig. 7A, *n*=6) along with the CX activity before and after venom injection. In parallel with CX activity, motoneuron activity followed the trend of decreasing activity after venom injection (Fig. 7A). We estimated normalized CX activity (Fig. 7B, red) and normalized leg motoneuron activity (Fig. 7B blue). Our results show a decrease of up to 80% in the normalized CX activity immediately after the venom injection (Fig. 7B). However, this decrease in firing rate was transient and lasted ∼10 min. Eventually, CX activity rebounded and remained elevated for the next 20 min, only to decrease again over time. In fact, after 2 h of venom injection, normalized CX activity regressed to ∼60% of the normalized baseline activity (Fig. 7B, red). Although long-term reduction of normalized leg motoneuron activity (Fig. 7B, blue) by wasp venom was comparable to its action on normalized CX activity, short-term effects are distinctly different. Immediately after venom injection, normalized motoneuron activity began to increase and peaked within 10 min of venom injection. Following this initial increase in activity, normalized motoneuron activity restabilized to a lower level and continued to fade away over time (Fig. 7B, blue). Furthermore, by quantifying the Ds activity (Fig. 7C), our results show that ∼90 min after venom injection, Ds activity decreased up to 60% compared with baseline level prior to the venom injection (Fig. 7C). Furthermore, we also established that a reduction in CX activity (*n*=9 units from 6 animal) significantly correlated with diminished leg motoneuron activity (*n*=6 units from 6 animals; Fig. 7D, Pearson correlation, *r*=0.5605, *P*=0.0463).

## DISCUSSION

Our results provide a neural basis for venom-induced long-term hypokinesia induced by *Ampulex* stings. A hallmark of hypokinesia is the absence of spontaneous walking (Fouad et al., 1994; Libersat, 2003). We show that hypokinesia correlates with an overall reduction in CX neuronal activity (Fig. 2). *Ampulex* venom is a rich cocktail of various proteins and peptides (Arvidson et al., 2019) that induce a multi-stage behavioral sequence, including grooming, and hypokinesia (Libersat, 2003). Our results revealed a distinctive pattern of CX neuronal firing after venom injection that can account for the onset of the behavioral manipulation. We found that neuronal activity in the CX decreases dramatically immediately after venom injection (Figs 2 and 7B). This reduction is sharp but short-lived, as normalized neuronal activity in the CX regresses by ∼80% but begins to rebound within 5 min (Fig. 7B). This instant reduction in the CX neuronal activity could be explained by the high concentration of GABA found in the wasp venom (Moore et al., 2006). A similar reduction in neuronal activity was reported after GABA injection into abdominal ganglia (Moore et al., 2006). However, after the initial silencing, the neuronal activity rebounds and remains elevated for the next 20–30 min. Interestingly, the timing of rebound neuronal activity matches the beginning and end of the grooming period after a natural sting (Weisel-Eichler et al., 1999). In addition to GABA, wasp venom contains dopamine (Banks and Adams, 2012; Moore et al., 2018; Weisel-Eichler et al., 1999), which is shown to induce intense and prolonged grooming behavior. A recent study shows that the D1-like dopamine receptor (dopR) is the venom target responsible for grooming evoked by the sting into the CX (Nordio et al., 2022). Furthermore, studies from *Drosophila* demonstrated that dopR receptors are highly expressed in the fly CX (Kong et al., 2010; Pitmon et al., 2016). Given these facts, it is possible that the rebound activity 10 min after venom injection could be associated with grooming behavior, which we are currently investigating. Subsequently, the rebound activity begins to wear off ∼30 min after venom injection and remains suppressed throughout the recording period of 2 h (Figs 2B and 7B). In fact, 2 h after venom injection, spontaneous activity of CX regresses to ∼60% of baseline activity. While the molecular mechanism of the silencing is yet to be discovered, it could be due to venom components interfering with synaptic transmission in the CX as proposed by Kaiser and colleagues (2019). This possibility becomes even more relevant with our results showing that wasp venom not only reduces the spontaneous activity of CX, but also modulates its ability to process sensory information.

Reduction in neuronal activity of the CX cannot be attributed to artefacts of the experimental procedure. To ensure that this is the case, we tested the viability of the preparation for chronic recordings by injecting procaine, a reversible Na^+^ channel inhibitor, into the CX using the same protocol as per venom injection protocol. As expected from a successful injection of this Na^+^ channel inhibitor, neuronal activity is blocked immediately after injection (Fig. 1) but recovers 50–60 min post-injection. It is important to point out that recovery of neuronal activity is robust; no significant difference was found 80 min after injection. Indeed, neuronal activity was statistically identical to baseline activity 90 min after procaine injection (*P*=1.000). Furthermore, the time course of recovery of CX activity is very similar to that of behavioral recovery after procaine injection into the CX described previously (Kaiser and Libersat, 2015). In addition to procaine injection, we also injected saline into the CX (Fig. S1). While procaine injection induces a reversible inhibition of the neuronal activity, no changes in activity were observed following saline injection. In the case of saline injection, CX neuronal activity not only remained the same as baseline at all the time intervals post-injection, but in fact, in most instances, the quality of recording improved over time (Fig. S1). Thus, our results from both procaine and saline injections rule out the possibility that either the short-term (immediately after venom injection) or long-term (∼2 h after injection) reduction in neuronal activity could be due to our experimental protocol. Furthermore, by tracking the activity of identified spike waveform before and after injection, we ruled out the possibility that reduction in the neuronal activity could be due to movement of the electrode (Fig. S2). Therefore, the complex pattern of CX firing rate that arises after the venom injection is very likely due to the wasp venom alone.

Reduction of neuronal activity owing to CX envenomation is not limited to cerebral ganglia and is paralleled by a decrease in DIN activity (Fig. 6A). We found that DIN activity recorded from neck connectives between the head ganglia and prothoracic ganglia in stung cockroaches was reduced up to 33% 4–6 h after the wasp sting as compared with activity in unstung counterparts (Fig. 6B). Reduction in DIN activity is both relevant and expected because the CX is one of the regions providing input to the DIN (Heinze and Homberg, 2008). Hence, venom-induced neuronal modulation in the CX has an impact on descending and permissive control of thoracic motor circuitries. It is worth mentioning that the hypokinesia-like state that begins after the grooming period lasts for 4–5 days (Fouad et al., 1994; Libersat, 2003). Throughout this period, very little change in behavior of stung cockroaches is reported. Thus, despite the difference in the recording time, comparison of both groups (CX and DIN) is relevant, as both time periods fall well within the already identified period of hypokinesia.

In an unstung cockroach, escape running involves activity of both slow (Ds) and a fast (Df) motoneurons innervating the coxal depressor muscle of the metathoracic legs (Watson and Ritzmann, 1998b,a). Ds alone is recruited in posture and slow walking (Emanuel et al., 2020; Watson and Ritzmann, 1998a), while the fast motor neuron (Df) is recruited in fast running and escape responses (Watson and Ritzmann, 1998b). Furthermore, although the primary target for the wasp sting to the brain is the CX, the ultimate consequence is reflected as suppression of Ds motor activity and the virtual absence of Df activity (Emanuel and Libersat, 2017). In addition, Emanuel and Libersat (2017) found that Ds motoneuron activity in the coxal muscle of the metathoracic leg is reduced drastically with procaine injection into the CX (Emanuel and Libersat, 2017). In the present study, we found that after venom injection, Ds activity decreased up to 60% compared with baseline level prior to the venom injection (Fig. 7C). This reduction in Ds motor activity is comparable to the reduction of CX activity, which is diminished by ∼70% from baseline levels over the same time frame (Fig. 2B; Table S2). It is established that reduction of Ds tonic activity is a hallmark feature of venom-induced hypokinesia (Emanuel and Libersat, 2017). Furthermore, reduction of CX neuronal activity is found to be positively correlated with reduction in Ds activity during the 2 h recording window (Fig. 7D). Thus, although we cannot prove causality, the results reported in the present study provide compelling evidence that loss of spontaneous walking and induction of the venom-induced behavioral sequence leading to hypokinesia is likely due to modulation of a feedforward descending pathway from the CX to thoracic motor circuitries. Compromised CX activity leads to reduction in tonic permissive traffic from the brain to the thoracic ganglia. Diminished activity of thoracic ganglia is associated with reduced excitability of both Ds and Df motoneurons, which ultimately leads to suppressed spontaneous walking (Emanuel and Libersat, 2017; Fouad et al., 1996).

Once the effect of wasp venom on spontaneous activity was established, we focused our attention on how it influences stimulus-evoked activity. Besides being the pre-motor center in the cockroach brain (Bender et al., 2010; Guo and Ritzmann, 2013), the CX is also a multi-modal processing center that has roles in azimuth calculation, processing of information related to polarized light and responses to light stimuli (Heinze and Homberg, 2007; Heinze et al., 2009; Homberg et al., 2011; Ritzmann et al., 2008). Thus, to evaluate the effect of wasp venom on the ability of CX to process sensory information, we tested evoked responses to blue light stimuli using a custom-built platform with 5 blue LEDs placed 10 cm above the animal. We identified two distinct types of units responding to light stimuli. Light ON units initiate a response at onset of the stimulus that continues during the applied stimulus duration (Figs 3A and 4A), whereas light OFF units respond after the stimulus ends (Figs 3B and 4B). Intriguingly, our sampling appears to reveal that the population of light OFF units (*n*=24 units from 15 animals) is much larger than the light ON population (*n*=8 units from 5 animals). This result seems consistent with the fact that cockroaches are nocturnal beings with high mobility in the dark. Our results indicate that duration of responses triggered by light stimuli varies depending on the type of cell (light ON or light OFF) and state of the animal (stung versus unstung). Before venom injection, light ON unit activity increases and remains elevated for the entire 500 ms stimulus duration as compared with the baseline (Figs 3A and 4A). In contrast, the total duration of light OFF responses is ∼200 ms following end of the stimulus (Figs 3B and 4B). However, venom injection into the CX does not produce the same effect in both types of light-responsive units. Light OFF unit response duration remains unchanged (200 ms). In contrast, light ON unit response duration is essentially eliminated. In addition to effects on response duration, venom injection into the CX causes reduction in evoked activity for both types of light-responsive units. In the case of light OFF units, a pairwise comparison of neuronal activity before and after venom injection for each time bin reveals that evoked average responses shrink to almost half of pre-venom injection levels (Fig. 4B). Similarly, light ON units exhibit very weak or no activity after venom injection and evoked average responses are not significantly elevated in any time bin following the stimulus onset (Figs 3A and 4A). While evoked responses seem to point to neuronal suppression due to envenomation of CX, more direct evidence of the effect of wasp venom on evoked neuronal activity arises when analyzing total evoked activity before and after venom injection. Total evoked activity analysis reveals an 80% decrease in responses of light ON units after venom injection (Fig. 5C). It is worth noting that this difference is due to the diminished ability of light ON units to elicit any response to light stimuli after venom injection (Fig. 3A). By comparison, a 45% decrease in the total evoked activity is reported for light OFF units (Fig. 5D). Likewise, our results reveal a reduction in peak neuronal responses for both types of light-responsive units. For light ON units, peak neuronal responses decrease by more than 50% after venom injection (Fig. 5A, right; *n*=8 units from 5 animals) as compared with their responses before the venom injection (Fig. 5A, left; *n*=8 units from 5 animals). Similarly, for light OFF units, peak neuronal responses decrease up to ∼30% after venom injection (*n*=24 units from 15 animals) as compared with responses prior to venom injection. Thus, after venom injection, suppression of the light-evoked response is found to be consistent for a variety of parameters. This is not the first instance where wasp venom is found to modulate the sensory-evoked activity in the CX. For instance, the wasp venom desensitizes the CX response to nociceptive stimuli in similar ways (Rana et al., 2022). Furthermore, venom injection into the CX reduces speed of sensory information processing, as response latency increases after venom injection regardless of the stimulus type. While this trend of increase in response latency has been reported for both light-responsive units, this increase is found to be significant only in light OFF units (graph not shown). In summary, wasp venom affects both evoked and spontaneous activity in the CX. This presumably ensures effectiveness of venom-induced hypokinesia as a subjugation strategy designed to make the cockroach a compliant food source.

### Conclusion

Previous studies have established the CX as a pre-motor command center involved in ongoing regulation of locomotion. It has roles in initiation, maintenance and termination of walking (Bender et al., 2010; Guo and Ritzmann, 2013; Martin et al., 1999; Pfeiffer and Homberg, 2014; Strauss and Heisenberg, 1993). However, pharmacological studies extend the functional roles of the CX beyond locomotion, implicating its involvement in almost all the behavioral changes induced after the wasp sting. For example, pharmacological block of dopR receptors in the CX prior to the wasp sting prevents sting-induced grooming behavior (Nordio et al., 2022) without affecting spontaneous walking. Likewise, focal injection of opiate agonists mimics venom effects on spontaneous walking and the escape threshold, but does not affect grooming behavior. Thus, CX circuitry selectively encodes a variety of functions and behavior via diverse chemical signaling pathways involving neurotransmitters and neuropeptides (Banks and Adams, 2012; Kaiser et al., 2019; Moore et al., 2006, 2018; Weisel-Eichler et al., 1999). The jewel wasp and its venom must be tapping on these diverse receptors in the CX to manipulate cockroach behavior. Our findings help to unravel the cascade of events occurring in cockroach head ganglia after wasp venom injection. The first potential weapon deployed by the wasp is presumably GABA, which reduces neuronal activity of the CX sharply, though briefly. As the actions of GABA wear off, dopamine present in the wasp venom triggers intense and prolonged grooming (Nordio et al., 2022). Subsequently, the other proteins and peptides in the venom suppress neuronal activity in the CX (Arvidson et al., 2019), leading to reduced DIN influences on thoracic ganglia circuitry. Diminished activity of thoracic ganglia reduces the excitability of both Ds and Df motoneurons, ultimately leading to suppressed spontaneous walking and onset of hypokinesia. Future experiments will focus on specific relationships between venom components and their molecular targets to provide a more complete understanding of how manipulation of CX activity leads to changes in motor control.

## Supplementary Material

10.1242/jexbio.245252_sup1 Supplementary information
