# Supplementary material for: Parasitoid wasp venom re-programs host behavior through downmodulation of brain central complex activity and motor output
Source: J Exp Biol. 2023 Feb 13;226(3):jeb245252. doi: 10.1242/jeb.245252 (PMC10088415; doi:10.1242/jeb.245252)
Supplement: Supplementary information [file jexbio-226-245252-s1.pdf]

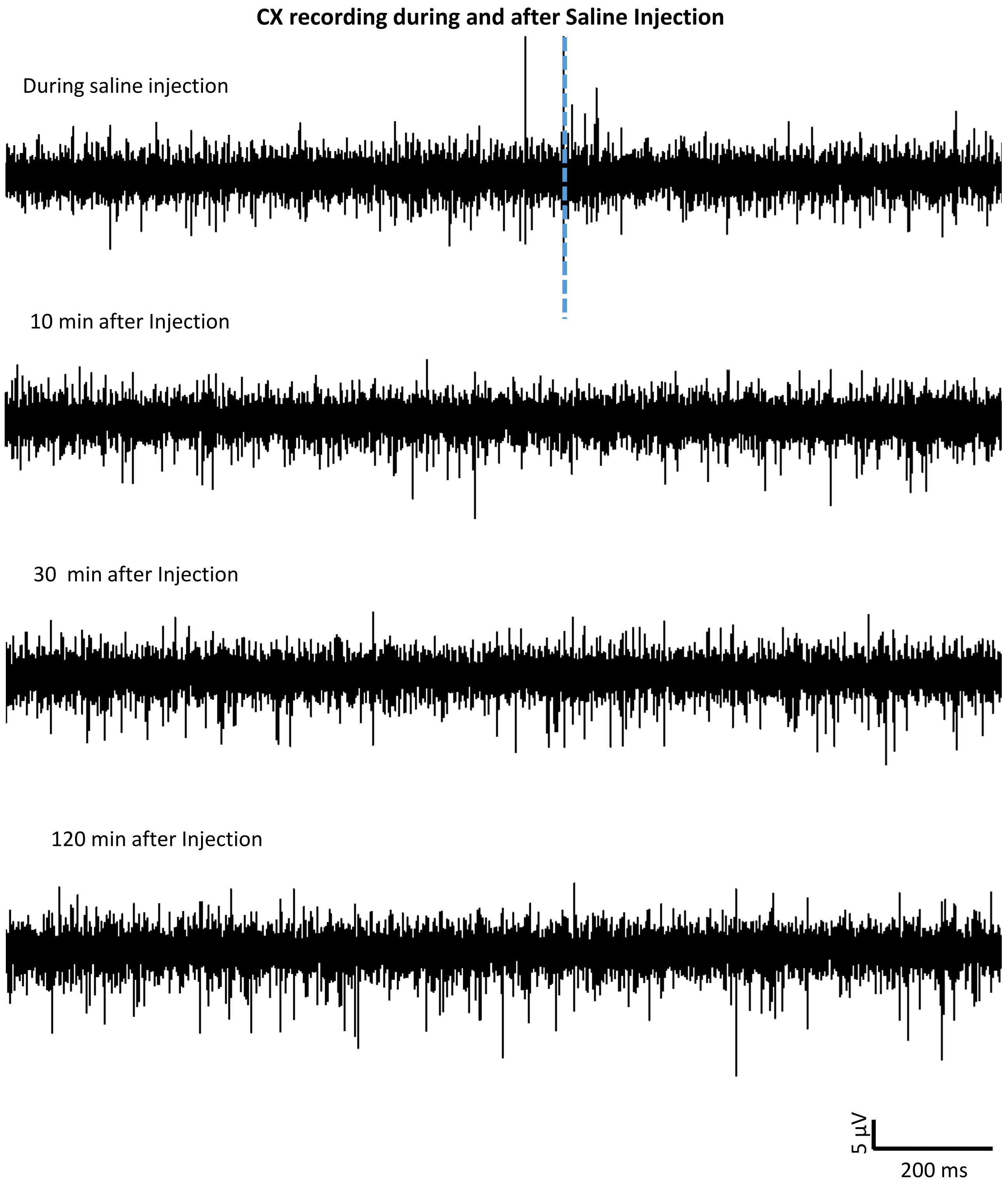

Fig. S1. Saline injection induces no change in CX activity: Representative examples of spontaneous neuronal activity in the CX at various times (10, 30, and 120 min after saline injection. Dashed blue bar represents the time of injection. CX units remain active throughout the two-hour long recording.

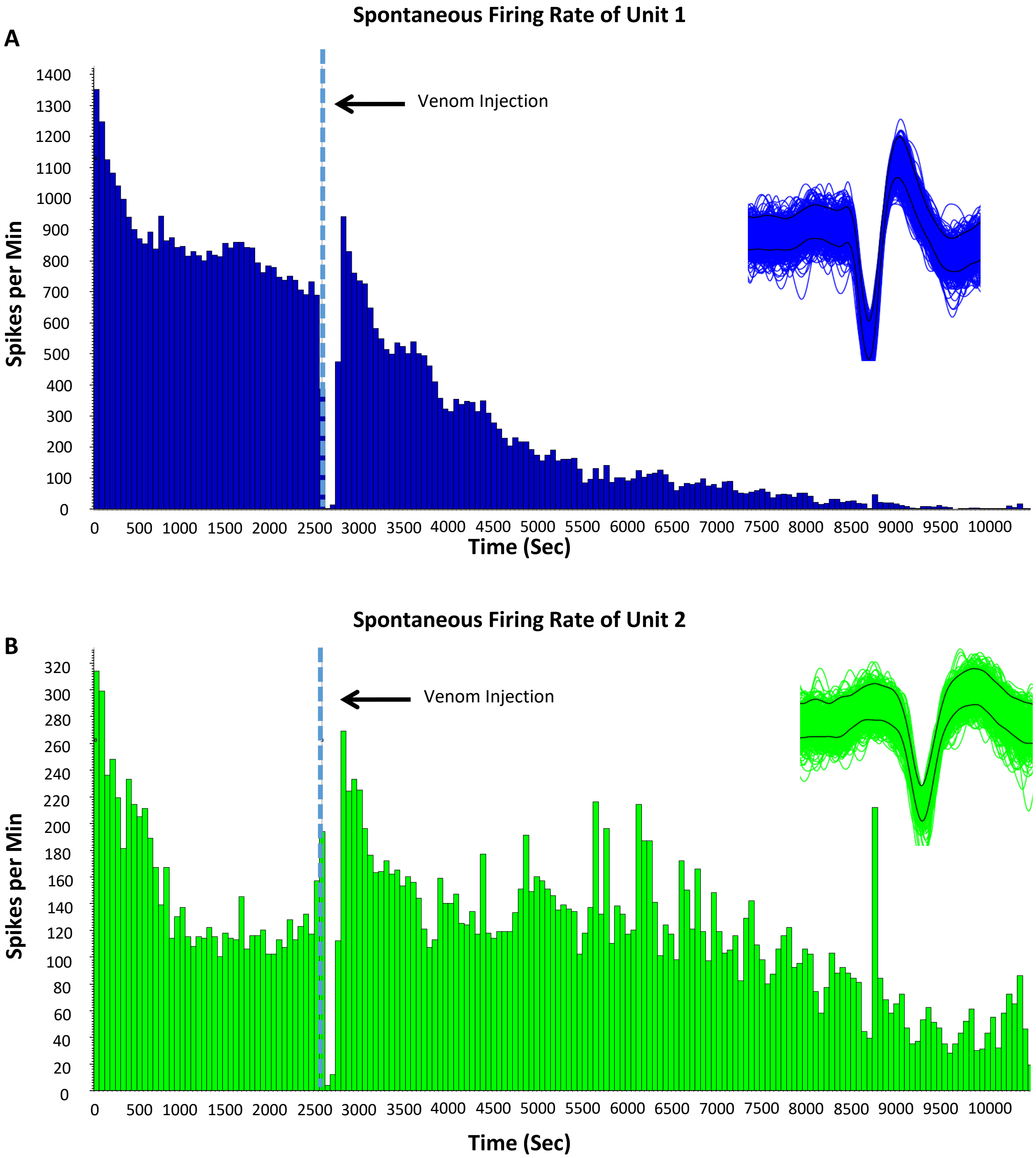

Fig. S2. Representative examples of neuronal firing rate of individually identified units from the same animal before and after venom injection along with the extracted waveform of each unit. The blue dashed line represents the time of venom injection in each case. (A) Spontaneous firing rate of identified unit 1; unit waveform is depicted in inset. The neuronal firing reduces greatly after the venom injection but recovers within 10 mins of injection. (B) Same as panel B for unit 2.

Table S1. Comparison of CX spontaneous activity before and after procaine injection

Time	Procaine Injection (n = 8 units from 5 animals)		
	Mean ± SEM Spike frequency (Hz)	T-value	P-Value (One Way repeated measures Anova, multiple comparison against control using Bonferroni t-test)
Baseline (Spontaneous Activity before Procaine injection)	7.92±2.22	-	-
10 min	0.15±0.04	5.019	<0.001
20 min	0.01±0.007	5.105	<0.001
30 min	0.02±0.01	5.098	<0.001
40 min	0.1±0.05	5.053	<0.001
50 min	0.31±0.18	4.913	<0.001
60 min	2.22±0.93	3.681	0.005
70 min	2.23±0.77	3.677	0.005
80 min	3.97±1.65	2.552	0.129*
90 min	5.5±1.6	1.565	1.000*
100 min	5.06±1.83	1.647	1.000*

- Values in the red font with an asterisk symbol represent non-significance in the P-value
- F-value 6.314

Journal of Experimental Biology • Supplementary information

Table S2. Comparison of CX spontaneous activity before and after venom injection

Time	Venom Injection (n = 35 units from 21 animals)		
	Mean ± SEM Spike frequency (Hz)	T-value	P-Value (One Way repeated measures Anova, multiple comparison against control using Bonferroni t-test)
Baseline (Spontaneous Activity before Venom injection)	6.32±1.65	-	-
10 min	2.89±0.48	3.433	<0.001
20 min	5.14±1.06	1.175	1.00*
30 min	5.24±1.03	1.078	1.00*
40 min	3.8±0.67	2.517	0.043
50 min	3.18±5.41	3.141	0.003
60 min	3.03±0.53	3.285	0.002
70 min	2.88±0.56	3.442	<0.001
80 min	2.65±0.59	3.665	<0.001
90 min	1.95±0.31	4.368	<0.001
100 min	1.63±0.22	4.721	<0.001
110 min	1.72±0.311	4.859	<0.001
120 min	1.43±0.37	5.113	<0.001

- Values in the red font with an asterisk symbol represent non-significance in the P-value.
- F-value = 6.125
